# 3-[3-(Pyridin-3-yl)-1,2,4-oxadiazol-5-yl]propanoic acid

**DOI:** 10.1107/S1600536810051639

**Published:** 2010-12-18

**Authors:** Fang Zhang, Fei Liu, Qifan Chen, Mingdong Dong

**Affiliations:** a College of Chemical Engineering & Materials, Eastern Liaoning University, No. 325 Wenhua Road, Yuanbao District, Dandong City, Liaoning Province 118003, People’s Republic of China; b Experiment Center, Eastern Liaoning University, No. 325 Wenhua Road, Yuanbao District, Dandong City, Liaoning Province 118003, People’s Republic of China

## Abstract

In the title compound, C_10_H_9_N_3_O_3_, the benzene ring is almost coplanar with the heterocyclic ring, making a dihedral angle of 11.3 (1)°. The plane of the carboxyl group is rotated by 8.4 (2)° with respect to the 1,2,4-oxadiazole ring plane. The aliphatic chain exhibits an extended conformation. In the crystal, mol­ecules are liked through inter­molecular O—H⋯N bonds, forming a chain structure along the *c* axis.

## Related literature

For the biological activity of 1,2,4-oxadiazo­les, see: Jakopin & Dolenc, 2008 ▶). For the use of this heterocycle as a core for luminescent liquid crystals, see: Gallardo *et al.* (2008 ▶). For related structures, see: Santos *et al.* (2009 ▶); Wang *et al.* (2006 ▶, 2007 ▶)
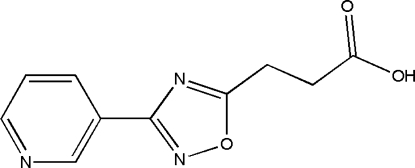


## Experimental

### 

#### Crystal data

C_10_H_9_N_3_O_3_
*M* *_r_* = 219.20
Orthorhombic, 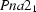

*a* = 6.1298 (12) Å
*b* = 6.8194 (14) Å
*c* = 23.426 (5) Å
*V* = 979.2 (3) Å^3^
*Z* = 4
Mo *K*α radiation
μ = 0.11 mm^−1^
*T* = 293 K
0.33 × 0.22 × 0.21 mm

#### Data collection

Siemens P4 diffractometer
8147 measured reflections
1138 independent reflections
884 reflections with *I* > 2σ(*I*)
*R*_int_ = 0.049

#### Refinement

*R*[*F* ^2^ > 2σ(*F* ^2^)] = 0.034
*wR*(*F* ^2^) = 0.083
*S* = 1.00
1138 reflections
146 parameters
1 restraint
H-atom parameters constrained
Δρ_max_ = 0.14 e Å^−3^
Δρ_min_ = −0.16 e Å^−3^

### 

Data collection: *XSCANS* (Bruker, 2003 ▶); cell refinement: *XSCANS*; data reduction: *XSCANS* and *SHELXTL* (Sheldrick, 2008 ▶); program(s) used to solve structure: *SHELXS97* (Sheldrick, 2008 ▶); program(s) used to refine structure: *SHELXL97* (Sheldrick, 2008 ▶); molecular graphics: *SHELXTL*; software used to prepare material for publication: *SHELXTL*.

## Supplementary Material

Crystal structure: contains datablocks global, I. DOI: 10.1107/S1600536810051639/fk2032sup1.cif

Structure factors: contains datablocks I. DOI: 10.1107/S1600536810051639/fk2032Isup2.hkl

Additional supplementary materials:  crystallographic information; 3D view; checkCIF report

## Figures and Tables

**Table 1:** Hydrogen-bond geometry (Å, °)

*D*—H⋯*A*	*D*—H	H⋯*A*	*D*⋯*A*	*D*—H⋯*A*
O1—H1⋯N3^i^	0.82	1.89	2.704 (3)	172

Symmetry code: (i) 
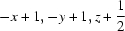
.

**Fig. 1.**
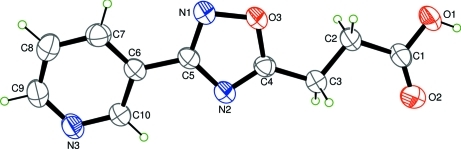
Structure of the title compound showing 50% probability displacement ellipsoids.

## supplementary crystallographic information

### Comment

1,2,4-Oxadiazoles are well known compounds, which exhibit a large number of biological activities (Jakopin & Dolenc, 2008). Recently, the use of this heterocycle as core for luminescent liquid crystals has also been described (Gallardo *et al.*, 2008). Here we report the structure of title compound (Fig. 1), the benzene ring is almost coplanar with the heterocyclic ring, making a dihedral angle of 11.3 (1) °. The torsion angle N2—C5—C6—C10 between the pyridine ring attached to C-5 of the 1,2,4-oxadiazole system is -8.0 (3) °, both rings are almost coplanar. The C-4 side-chain containing a carboxylic acid group shows a zigzag arrangement, having the torsion angle C1—C2—C3—C4 of -178.0 (2) °. In addition, the plane of the carboxylic group is also rotated by 8.4 (2) ° with respect to the mean plane of the 1,2,4-oxadiazole five-membered ring. This makes the molecular structure to be slightly twisted. In the crystal structure, molecules are liked through intermolecular O—H···N, forming a one-dimensional chain structure along the crystallographic *c* axis.

### Experimental

To a solution of nitrile (0.2 mol) in ethanol (20 mL) was added hydroxylamine hydrochloride (0.4 mol) in water (40 mL). Then anhydrous sodium carbonate(0.4 mol) in water (120 mL) was slowly added to the resulting solution and the mixture was stirred at 358k for 5 h. The mixture was then concentrated under vacuum to evaporate some water. The resulting suspension was filtered, the amidoxime solid formed was washed with cold water, dried under vacuum. A thoroughly triturated mixture of amidoxime (0.04 mol) and succinic anhydride (0.08 mol) was heated in an oily bath to 403k and kept at this temperature for 4 h. The reaction mixture was cooled to room temperature, and the product was washed with cold water, filtered, and recrystallized from ethanol. Block-shaped crystals suitable for X-ray diffraction were obtained from methanol.

### Refinement

H atoms bound to C atoms were placed in calculated positions and treated as riding on their parent atoms, with C—H = 0.93 Å (aromatic C), C—H = 0.97Å (methylene C), and with *U*_iso_(H) = 1.2U_eq_(C). The H atom bound to O1 was located from Fourier difference map and refined with O—H = 0.82 Å, *U*_iso_(H) = 1.5U_eq_(O).

### Crystal data

**Table:**

C_10_H_9_N_3_O_3_	*F*(000) = 456
*M**_r_* = 219.20	*D*_x_ = 1.487 Mg m^−^^3^
Orthorhombic, *P**n**a*2_1_	Mo *K*α radiation, λ = 0.71073 Å
Hall symbol: P 2c -2n	Cell parameters from 6383 reflections
*a* = 6.1298 (12) Å	θ = 3.1–27.5°
*b* = 6.8194 (14) Å	µ = 0.11 mm^−^^1^
*c* = 23.426 (5) Å	*T* = 293 K
*V* = 979.2 (3) Å^3^	Block, colourless
*Z* = 4	0.33 × 0.22 × 0.21 mm

### Data collection

**Table:**

Bruker P4 diffractometer	884 reflections with *I* > 2σ(*I*)
Radiation source: fine-focus sealed tube	*R*_int_ = 0.049
graphite	θ_max_ = 27.5°, θ_min_ = 3.1°
ω scans	*h* = −6→7
8147 measured reflections	*k* = −8→8
1138 independent reflections	*l* = −30→30

### Refinement

**Table:**

Refinement on *F*^2^	Primary atom site location: structure-invariant direct methods
Least-squares matrix: full	Secondary atom site location: difference Fourier map
*R*[*F*^2^ > 2σ(*F*^2^)] = 0.034	Hydrogen site location: geom and difmap
*wR*(*F*^2^) = 0.083	H-atom parameters constrained
*S* = 1.00	*w* = 1/[σ^2^(*F*_o_^2^) + (0.0487*P*)^2^] where *P* = (*F*_o_^2^ + 2*F*_c_^2^)/3
1138 reflections	(Δ/σ)_max_ < 0.001
146 parameters	Δρ_max_ = 0.14 e Å^−^^3^
1 restraint	Δρ_min_ = −0.16 e Å^−^^3^

### Special details

**Table:**

Geometry. All e.s.d.'s (except the e.s.d. in the dihedral angle between two l.s. planes) are estimated using the full covariance matrix. The cell e.s.d.'s are taken into account individually in the estimation of e.s.d.'s in distances, angles and torsion angles; correlations between e.s.d.'s in cell parameters are only used when they are defined by crystal symmetry. An approximate (isotropic) treatment of cell e.s.d.'s is used for estimating e.s.d.'s involving l.s. planes.
Refinement. Refinement of *F*^2^ against ALL reflections. The weighted *R*-factor *wR* and goodness of fit *S* are based on *F*^2^, conventional *R*-factors *R* are based on *F*, with *F* set to zero for negative *F*^2^. The threshold expression of *F*^2^ > σ(*F*^2^) is used only for calculating *R*-factors(gt) *etc*. and is not relevant to the choice of reflections for refinement. *R*-factors based on *F*^2^ are statistically about twice as large as those based on *F*, and *R*- factors based on ALL data will be even larger.

### Fractional atomic coordinates and isotropic or equivalent isotropic displacement parameters (Å^2^)

**Table:**

	*x*	*y*	*z*	*U*_iso_*/*U*_eq_	
O3	0.8809 (3)	0.4377 (3)	0.16313 (7)	0.0450 (5)	
O1	0.4239 (4)	0.4072 (4)	0.33554 (8)	0.0628 (6)	
H1	0.3237	0.4277	0.3579	0.094*	
O2	0.2140 (4)	0.5819 (3)	0.27757 (9)	0.0681 (7)	
N3	0.8817 (4)	0.5436 (3)	−0.08242 (9)	0.0468 (6)	
C10	0.8297 (5)	0.5499 (4)	−0.02742 (11)	0.0412 (6)	
H10	0.6895	0.5885	−0.0173	0.049*	
N2	0.6960 (4)	0.5622 (3)	0.09157 (9)	0.0394 (5)	
N1	1.0181 (4)	0.4195 (3)	0.11489 (9)	0.0461 (5)	
C6	0.9757 (4)	0.5012 (4)	0.01552 (11)	0.0358 (5)	
C5	0.8992 (4)	0.4978 (3)	0.07499 (10)	0.0346 (5)	
C1	0.3807 (5)	0.4921 (4)	0.28656 (12)	0.0430 (6)	
C2	0.5595 (4)	0.4565 (4)	0.24436 (11)	0.0426 (6)	
H2A	0.5711	0.3167	0.2373	0.051*	
H2B	0.6967	0.5002	0.2606	0.051*	
C3	0.5225 (5)	0.5608 (4)	0.18853 (10)	0.0427 (6)	
H3A	0.3826	0.5204	0.1731	0.051*	
H3B	0.5153	0.7008	0.1956	0.051*	
C8	1.2425 (5)	0.4437 (4)	−0.05639 (12)	0.0491 (7)	
H8	1.3826	0.4088	−0.0677	0.059*	
C7	1.1881 (4)	0.4503 (4)	0.00047 (12)	0.0449 (6)	
H7	1.2912	0.4214	0.0284	0.054*	
C4	0.6937 (4)	0.5226 (3)	0.14541 (10)	0.0358 (5)	
C9	1.0850 (5)	0.4898 (4)	−0.09637 (13)	0.0497 (7)	
H9	1.1220	0.4832	−0.1348	0.060*	

### Atomic displacement parameters (Å^2^)

**Table:**

	*U*^11^	*U*^22^	*U*^33^	*U*^12^	*U*^13^	*U*^23^
O3	0.0460 (11)	0.0605 (10)	0.0285 (9)	0.0084 (8)	−0.0026 (8)	0.0037 (8)
O1	0.0615 (14)	0.0953 (16)	0.0315 (10)	0.0191 (11)	0.0058 (9)	0.0162 (10)
O2	0.0625 (15)	0.0933 (16)	0.0484 (13)	0.0305 (12)	0.0078 (11)	0.0187 (11)
N3	0.0530 (14)	0.0543 (12)	0.0331 (12)	−0.0001 (10)	0.0009 (10)	0.0043 (10)
C10	0.0439 (18)	0.0467 (13)	0.0329 (13)	0.0034 (12)	−0.0010 (11)	0.0005 (11)
N2	0.0409 (11)	0.0462 (11)	0.0311 (11)	0.0030 (9)	−0.0008 (10)	0.0040 (9)
N1	0.0466 (12)	0.0580 (14)	0.0336 (11)	0.0079 (10)	0.0017 (10)	0.0048 (9)
C6	0.0411 (14)	0.0344 (10)	0.0319 (12)	−0.0020 (11)	−0.0028 (11)	0.0010 (9)
C5	0.0388 (13)	0.0340 (11)	0.0310 (12)	−0.0018 (11)	−0.0043 (11)	0.0008 (9)
C1	0.0473 (17)	0.0506 (14)	0.0311 (13)	−0.0015 (13)	−0.0036 (12)	0.0005 (10)
C2	0.0437 (15)	0.0525 (14)	0.0316 (13)	0.0027 (10)	−0.0015 (12)	0.0018 (11)
C3	0.0460 (15)	0.0503 (13)	0.0317 (13)	0.0066 (12)	0.0007 (12)	0.0021 (11)
C8	0.0450 (17)	0.0503 (16)	0.0521 (18)	−0.0005 (12)	0.0116 (14)	−0.0025 (12)
C7	0.0399 (15)	0.0470 (14)	0.0479 (17)	0.0019 (12)	−0.0022 (13)	0.0039 (12)
C4	0.0382 (14)	0.0361 (11)	0.0332 (14)	0.0011 (10)	−0.0050 (10)	−0.0013 (10)
C9	0.0575 (19)	0.0544 (15)	0.0371 (15)	−0.0021 (14)	0.0090 (13)	0.0014 (12)

### Geometric parameters (Å, °)

**Table:**

O3—C4	1.351 (3)	C6—C5	1.470 (4)
O3—N1	1.414 (3)	C1—C2	1.496 (4)
O1—C1	1.312 (3)	C2—C3	1.506 (3)
O1—H1	0.8200	C2—H2A	0.9700
O2—C1	1.210 (3)	C2—H2B	0.9700
N3—C10	1.328 (4)	C3—C4	1.480 (3)
N3—C9	1.340 (4)	C3—H3A	0.9700
C10—C6	1.387 (4)	C3—H3B	0.9700
C10—H10	0.9300	C8—C7	1.374 (4)
N2—C4	1.290 (3)	C8—C9	1.381 (4)
N2—C5	1.377 (3)	C8—H8	0.9300
N1—C5	1.300 (3)	C7—H7	0.9300
C6—C7	1.393 (4)	C9—H9	0.9300
			
C4—O3—N1	107.29 (18)	C3—C2—H2B	109.0
C1—O1—H1	109.5	H2A—C2—H2B	107.8
C10—N3—C9	117.9 (2)	C4—C3—C2	113.7 (2)
N3—C10—C6	122.8 (3)	C4—C3—H3A	108.8
N3—C10—H10	118.6	C2—C3—H3A	108.8
C6—C10—H10	118.6	C4—C3—H3B	108.8
C4—N2—C5	102.6 (2)	C2—C3—H3B	108.8
C5—N1—O3	101.84 (19)	H3A—C3—H3B	107.7
C10—C6—C7	118.6 (2)	C7—C8—C9	118.7 (3)
C10—C6—C5	119.1 (2)	C7—C8—H8	120.6
C7—C6—C5	122.3 (2)	C9—C8—H8	120.6
N1—C5—N2	115.8 (2)	C8—C7—C6	118.7 (2)
N1—C5—C6	120.6 (2)	C8—C7—H7	120.6
N2—C5—C6	123.4 (2)	C6—C7—H7	120.6
O2—C1—O1	123.1 (3)	N2—C4—O3	112.4 (2)
O2—C1—C2	125.9 (3)	N2—C4—C3	129.6 (3)
O1—C1—C2	111.0 (2)	O3—C4—C3	117.9 (2)
C1—C2—C3	112.8 (2)	N3—C9—C8	123.2 (3)
C1—C2—H2A	109.0	N3—C9—H9	118.4
C3—C2—H2A	109.0	C8—C9—H9	118.4
C1—C2—H2B	109.0		
			
C9—N3—C10—C6	−0.8 (4)	O1—C1—C2—C3	−176.8 (2)
C4—O3—N1—C5	1.1 (2)	C1—C2—C3—C4	−178.1 (2)
N3—C10—C6—C7	2.4 (4)	C9—C8—C7—C6	0.7 (4)
N3—C10—C6—C5	−175.4 (2)	C10—C6—C7—C8	−2.3 (4)
O3—N1—C5—N2	−1.3 (3)	C5—C6—C7—C8	175.4 (2)
O3—N1—C5—C6	−176.9 (2)	C5—N2—C4—O3	−0.2 (3)
C4—N2—C5—N1	1.0 (3)	C5—N2—C4—C3	178.2 (2)
C4—N2—C5—C6	176.4 (2)	N1—O3—C4—N2	−0.6 (3)
C10—C6—C5—N1	167.5 (2)	N1—O3—C4—C3	−179.1 (2)
C7—C6—C5—N1	−10.2 (3)	C2—C3—C4—N2	168.1 (2)
C10—C6—C5—N2	−7.7 (3)	C2—C3—C4—O3	−13.6 (3)
C7—C6—C5—N2	174.6 (2)	C10—N3—C9—C8	−0.9 (4)
O2—C1—C2—C3	5.1 (4)	C7—C8—C9—N3	0.9 (4)

### Hydrogen-bond geometry (Å, °)

**Table:**

*D*—H···*A*	*D*—H	H···*A*	*D*···*A*	*D*—H···*A*
O1—H1···N3^i^	0.82	1.89	2.704 (3)	172

Symmetry codes: (i) −*x*+1, −*y*+1, *z*+1/2.
